# Clinical decision modeling system

**DOI:** 10.1186/1472-6947-7-23

**Published:** 2007-08-13

**Authors:** Haiwen Shi, James Lyons-Weiler

**Affiliations:** 1 Bioinformatics Analysis Core, Genomics and Proteomics Core Laboratories, 3343 Forbes Avenue, Pittsburgh, PA 15260 USA; 2 Department of Biomedical Informatics, University of Pittsburgh Medical School and University of Pittsburgh Graduate School of Public Health, Parkvale Building M-183, 200 Meyran Avenue, Pittsburgh, PA 15260 USA; 3 Department of Pathology, University of Pittsburgh, School of Medicine, S-417 BST, 200 Lothrop Street, Pittsburgh, PA 15261 USA; 4 Clinical Genomics Facility and Clinical Proteomics Facility, University of Pittsburgh Cancer Institute, Hillman Cancer Center, UPCI Research Pavilion, Suite 2.26d, 5177 Centre Ave., Pittsburgh, PA 15213-1863, USA; 5 Interdisciplinary Biomedical Graduate Program, University of Pittsburgh, School of Medicine Graduate Office, 524 Scaife Hall, Pittsburgh, PA 15261-0001 USA; 6 University of Pittsburgh Cancer Institute, 5150 Centre Ave, Pittsburgh, PA 15232, USA

## Abstract

**Background:**

Decision analysis techniques can be applied in complex situations involving uncertainty and the consideration of multiple objectives. Classical decision modeling techniques require elicitation of too many parameter estimates and their conditional (joint) probabilities, and have not therefore been applied to the problem of identifying high-performance, cost-effective combinations of clinical options for diagnosis or treatments where many of the objectives are unknown or even unspecified.

**Methods:**

We designed a Java-based software resource, the Clinical Decision Modeling System (CDMS), to implement Naïve Decision Modeling, and provide a use case based on published performance evaluation measures of various strategies for breast and lung cancer detection. Because cost estimates for many of the newer methods are not yet available, we assume equal cost. Our use case reveals numerous potentially high-performance combinations of clinical options for the detection of breast and lung cancer.

**Results:**

Naïve Decision Modeling is a highly practical applied strategy which guides investigators through the process of establishing evidence-based integrative translational clinical research priorities. CDMS is not designed for clinical decision support. Inputs include performance evaluation measures and costs of various clinical options. The software finds trees with expected emergent performance characteristics and average cost per patient that meet stated filtering criteria. Key to the utility of the software is sophisticated graphical elements, including a tree browser, a receiver-operator characteristic surface plot, and a histogram of expected average cost per patient. The analysis pinpoints the potentially most relevant pairs of clinical options ('critical pairs') for which empirical estimates of conditional dependence may be critical. The assumption of independence can be tested with retrospective studies prior to the initiation of clinical trials designed to estimate clinical impact. High-performance combinations of clinical options may exist for breast and lung cancer detection.

**Conclusion:**

The software could be found useful in simplifying the objective-driven planning of complex integrative clinical studies without requiring a multi-attribute utility function, and it could lead to efficient integrative translational clinical study designs that move beyond simple pair wise competitive studies. Collaborators, who traditionally might compete to prioritize their own individual clinical options, can use the software as a common framework and guide to work together to produce increased understanding on the benefits of using alternative clinical combinations to affect strategic and cost-effective clinical workflows.

## Background

Classical decision analysis is a well-established field of primarily theoretical analytical inquiry that can shed light either on the optimality of decision in the face of complexity and uncertainty, or a series of decisions for a particular circumstance. Less commonly, it may be used to define a fixed protocol of options to follow as general guidelines. Decision trees are sometimes represented as bifurcating structures where each node represents a particular decision, and the internodes represent paths to secondary decision nodes. Most decision modeling to date in medicine has focused on the problem of identifying optimal decisions of use of new healthcare technology when confronted with alternative (usually mutually exclusive) healthcare interventions. For a recent methodological reviews focused on methods see [1] Philips et al., 2004, and for an overview of methods and criteria for quality assessment of decision modeling see [2] Weinstein, 2006.

Model inputs are usually risk preferences derived via expert elicitation (e.g., [3], Alberdi et al., 2004). In advanced decision modeling, all possible decision trees are represented as a single tree, and algorithms exist (e.g., roll-forward, roll-back) to define an optimal decision path based on the consideration of multiple objectives, the cost and benefit of which are ideally expressed as a common utility function. For a fully enumerated decision analysis, the full joint probability matrix should ideally be specified, but is rarely available, in which case uncertainty can be explored via sensitivity analysis.

In application, decision analysis and decision modeling are often used to develop computer-aided decision support systems within a particular field of biomedical specialization (e.g., radiology). They have rarely been used in studying or defining research priorities for integration of diverse clinical options, or for the study of the integration of new clinical options into existing clinical workflows. The reasons for the lack of advances in modeling integration are practical; modeling clinician-patient dyad preferences ('expert elicitation') is extremely hard, and among-site variance in preferences is high. Models have been proposed that elicit input from both patients and caregivers ([4] Col, 2005).

How to weigh the same evidence varies from individual to individual. Moreover, the reasoning used to render a particular decision or risk preferences may not in some cases be represented accurately as an easily defined model. Defining a useful common 'currency' in which the cost functions all considerations can be expressed in terms of a utility function can be difficult, especially when many variables influencing decisions must be considered. The construction of multi-attribute utility functions, except in their simplest form, is an arduous process in which few decision makers are willing to participate. Finally, collecting a sufficient amount of data and uniform preferences on all pairs of diverse proposed clinical options becomes intractable, especially when many or newly proposed clinical options are considered.

Djulbegovic et al (2000; [5]) show how evidence-based medicine (EBM) summary measures derived from population studies can be incorporated into the framework of clinical decision analysis. Such approaches are imminently useful in the goal of clinical decision making with available clinical options. This area is called "clinical decision support" for which numerous academic and commercial resources already exist. In contrast, our focus on clinical decision modeling is for when too many new clinical options have been proposed, as in the case of putative biomarkers for disease detection, and no clear route exists to establishing priorities for integrative evaluative and translational research to determine which combinations of clinical options might receive priority for further research as an integrated set of options within a clinical workflow.

As an aid to defining integrative translational research priorities, our goal is not clinical decision support *per se*; instead, our goal is to provide a framework for the rationale discussion for clinical research's impact of integrating diverse sources of clinical information. By providing such an underpinning for these discussions, useful and cost-effective combinations can be overtly explored while other, more costly or less effective combinations can be given lower priority. Our motivation is well-founded; indeed, in application, a recent study found that as the complexity of decisions made increases, the use of decision support systems decrease [6]. Thus, the use of classical decision analysis to effect integrative translational research seems unlikely at worst, and challenging at best (but see [7] Leal et al., 2007 for a practical computing resource that may yield possible exceptions).

We have devised an alternative strategy that we call "naïve decision modeling" (NDM) that accepts the intractability of deriving a fully defined model. Beginning with the most basic elements of risks associated with individual decision options (performance characteristics of clinical options), NDM requires a critically operational, but ultimately testable, assumption of conditional independence among successive clinical options.

It is assumed that the aim of the research enabled by NDM is to define a clinical workflow that integrates a high-performance, cost-effective decision tree for diagnostics that uses ruling-in and ruling-out assays. NDM is not designed for real-time, i.e., dynamic, clinical decision making (e.g., [8], Housset & Junod, 2003), but rather to derive a general decision tree to be studied as a potential (hypothetical) clinical workflow, with follow-up testing being specified by the outcome (+/-) of the previous test. NDM is designed to facilitate the clinical research study designs needed to establish cost-effective integrated standard-of-care clinical options.

In the first step of NDM, performance evaluation measures of individual clinical options are collected. In the second step, alternative hypothetical combinations of clinical options are then characterized based on their expected performance and cost or any other attribute that can be specified. The resulting combinations are rank-sorted by performance or cost, and then explored manually by experts (e.g., clinicians), who might reject specific combinations of clinical options as unlikely (e.g., unethical) hypothetical clinical workflows. Information on critical pairs of clinical options is derived during the second step. In a third step, the assumption of conditional dependence among critical pairs of clinician-selected clinical options should be tested with empirical (e.g., retrospective) data. The model is then determined to either meet the assumption of conditional independence, or to violate it. If a hypothetical combination is found that meets the assumption of conditional dependence, then further clinical study of that particular combination as fixed clinical workflow may be warranted. If the assumption of conditional independence is violated, then the model may be updated, including estimates of conditional probabilities, from the retrospective study, and the one particular hypothetical workflow re-assessed on the basis of the new information. A new search that uses the revised input can then also be conducted to identify new workflows that may, or may not, be superior to the previously selected near-optimal workflow.

In this paper, we describe our software resource, CDMS, which implements this evidence-based strategy to decision modeling to promote collaborative integrative translational clinical research.

## Implementation

CDMS is a standalone application with a user-friendly graphic interface. Both the application and its interface are implemented in Java. CDMS is provided as an executable jar file. To run it, the user should download a Java Runtime Environment Version 5.0 Update 6 or above. CDMS currently works under Windows XP and 2000 operating systems. However, since Java is a platform-independent language, the application would work under other Java-compatible operating systems as well.

### General implementation

CDMS requires a tab-delimited text file as input and a specific prevalence for the disease or condition being studied. After that, running CDMS is just as simple as pressing a button. All searching results are displayed in the CDMS interface graphically. In addition, CDMS creates two output files. The first files (*.cdms) contains all graphic results objects. The other file is a text output file that is used to record the complete searching details, which can be used in the future for results checking reference, for example, to repeat a previously saved search.

### Application features

#### Decision trees as hypothetical clinical workflows: representation & searching

CDMS uses a rooted bifurcating tree structure to represent a clinical workflow. In this representation, the first clinical option is applied to all patients in a clinical setting. At each node, the patient population is divided into test positive and test negative partitions, with subsequent follow-up testing or treatment indicated by subsequent nodes.

Searching among possible combinations is currently restricted to a random tree search algorithm [see Additional file 1]. CDMS searches randomly among possible tree topologies within a user-defined range of size (# of clinical options), and retains the "best" clinical workflows according to user-defined optimality constraints. The random search is strategic, for two reasons: first, near-optimal solutions may be more clinically realistic than computationally guaranteed optimal solutions, and second, an exhaustive search of all possible tree topologies is implausible for very large numbers of clinical options. Felsenstein (2004) [9] reports that there are 34,459,425 "rooted, bifurcating, labeled trees" for 10 nodes, 8,200,794,532,637,891,559,375 for 20 nodes, 4.9518 × 10^38 ^for 30 nodes, 1.00985 × 10^57 ^for 40 nodes, and 2.75292 × 10^76 ^for 50 nodes. Those numbers are based on assumption that the "left-right order of branching does not make any difference" (Felsenstein, 2004 [9]). However, this assumption does not hold for decision tree. Thus there are even more possible tree topologies. It is intractable to search all trees to find the global optimal decision tree given large numbers of clinical options. This strategy is undesirable anyway, as clinical researchers may reject the globally optimal tree as unrealistic or unethical.

The total number, and size of tree topologies searched is specified by the user through the *Control Panel *interface. In the future, various tree-searching heuristics and the branch-and-bound algorithm may be added, but it will be important to retain near-optimal trees as the main application of CDMS is to facilitate the visual exploration of alternative hypothetical cost-effective combinations of clinical options, and not necessarily to discover the set of globally optimal combinations.

#### Optimization by and reporting of expected performance characteristics

The retained results of a given search are presented in graphical and tabular form in various tabs. CDMS evaluates the performance of a tree or a clinical workflow by calculating its Emergent Expected Sensitivity (EESN), Emergent Expected Specificity (EESP), and Expected Overall Cost per Patient (EOCPP). The calculations of these terms are provided in the appendix A1 [see Additional file 1]. CDMS records the best performance tree topology found among all trees searched and presents it graphically in the *Optimal Tree Topology *tab. A summary of the results from a given tree search is also reported in the *TreeSearching Summary *tab. The summary includes the information such as the total number of tree topologies searched, number of tree topologies that satisfy the performance and/or cost constraints.

#### ROC Contour Plot tab

A contour plot is displayed in the ROC *Contour Plot *tab. The ROC Contour Plot displays the counts (frequencies) of the EESN and 1-EESP among all tree topologies searched based on their combined performance values of EESN and 1-EESP.

#### Cost Histogram tab

CDMS displays the average cost per patient distribution of all trees searched in a histogram. Both the contour plot and histogram can be displayed on logarithmic scales. The user can do this by mousing-over the legend and right-clicking the mouse button.

#### Tree Browser tab

Perhaps the most useful component of the output of CDMS is the *Tree Browser *tab. In the tree browser, the tree topologies that satisfy both performance and cost constraints are listed in the left side of the browser. Each tree topology is represented by its rank, its associated confusion matrix, and other specific scores such as its EESN, EESP, Emergent Expected Achieved Classification Error (EEACE), EOCPP, and tree size. The display includes a button to view the tree, and a button to reject a given tree. All rejected tree topologies are moved to the *Rejected Trees *sub tab, and can be restored from the rejected tree list.

#### Saving and re-loading saved results and print functions

The results from a particular search can be saved to disk to allow the user to retrieve and view them later. The user can print all the graphical objects displayed in the interface, including any displayed tree topology.

#### Input format

To use the software, the user must provide a tab-delimited text file of potential clinical options with SN, SP, and cost estimates for each option (Table 1 and Table 2). To make CDMS more flexible, any number of objectives can be added to the input file as additional columns. These additional columns are user-defined measures for each clinical option. An example would be 'time to test result' for each clinical option. Therefore, while the input file must contain at least 4 default columns, the input file may in fact contain more than 4 columns.

**Table 1:** The input values for clinical options for breast cancer detection derived from a literature search

***Proposed Diagnostic Tests/Assays***	***SN***	***SP***	***Cost($)***	***Reference***	***PMID***
Magnetic Resonance Imaging (MRI)	0.96	0.75	100.00	Imbriaco et al. 2001 [11]	11202453
Electrical Impedance Scanning	0.38	0.95	100.00	Stojadinovic et al., 2006 [12]	16491309
electronic resonance spectroscopy of albumin configuration	0.85	0.91	100.00	Seidel et al., 2005 [13]	16422355
SCM Test	0.81	0.85	100.00	Klein et al., 2002 [14]	14965713
Serum TSGF	0.6386	0.9089	100.00	Liang et al., 2002 [15]	12526228
mammaglobin (cutpoint 8.8)	0.688	0.888	100.00	Bernstein et al., 2005 [16]	16166429
SELDI (serum), CART, two surfaces	0.9	0.93	100.00	Vlahou et al., 2003 [17]	14499014
scintimammography on px w/suspicious breast mass	0.9	0.938	100.00	Polan et al., 2001 [18]	11353112
NAS bFGF, race, menopausal status + PSA	0.909	0.833	100.00	Hsiung et al., 2002 [19]	12184408
cytology alone, breast ductal lavage cells	0.67	0.93	100.00	Zhang et al., 2006 [20]	16477639
G-actin biomarker, breast ductal lavage cells	0.9	1	100.00	Zhang et al., 2006 [20]	16477639
DNA5cER biomarker, breast ductal lavage cells	1	0.93	100.00	Zhang et al., 2006 [20]	16477639

**Table 2:** The input values for clinical options for lung cancer detection derived from a literature and internet search

***CLINICAL OPTIONS***	***SN***	***SP***	***Cost($)***	**Reference**	***PMID***
TSGF	0.869	0.911	100.00	Newland Biotech [21]	N/A
NSE	0.4617	0.9323	100.00	Newland Biotech [21]	N/A
CA-125	0.5643	0.8472	100.00	Newland Biotech [21]	N/A
SELDI (serum)	0.869	0.8	100.00	Yang et al., 2005 [22]	16029516
TLP	0.667	0.8	100.00	Tarro et al., 2005 [23]	15389637
CA-19-9	0.333	1	100.00	Tarro et al., 2005 [23]	15389637
CYFRA 21-1	0.111	1	100.00	Tarro et al., 2005 [23]	15389637
CEA	0	1	100.00	Tarro et al., 2005 [23]	15389637
anti-Rc	0.2	0.98	100.00	Bazhin et al., 2004 [24]	15084384
LIFE bronchoscopy	0.68	0.696	100.00	Hirsch et al., 2001 [25]	11562389
WLB bronchoscopy	0.219	0.783	100.00	Hirsch et al., 2001 [25]	11562389
methylation in at least 1 of 5 tumor suppressor genes	0.495	0.85	100.00	Fujiwara et al., 2005 [26]	15709192
CT-guided fine-needle aspirate biopsy	0.82	1	100.00	Wallace et al., 2002 [27]	12461267
x-ray determinate only	0.54	0.99	100.00	Gavelli & Giampalma, 2000 [28]	11147625
x-ray indeterminate	0.84	0.9	100.00	Gavelli & Giampalma, 2000 [28]	11147625

#### Control Panel

**Figure 1:**
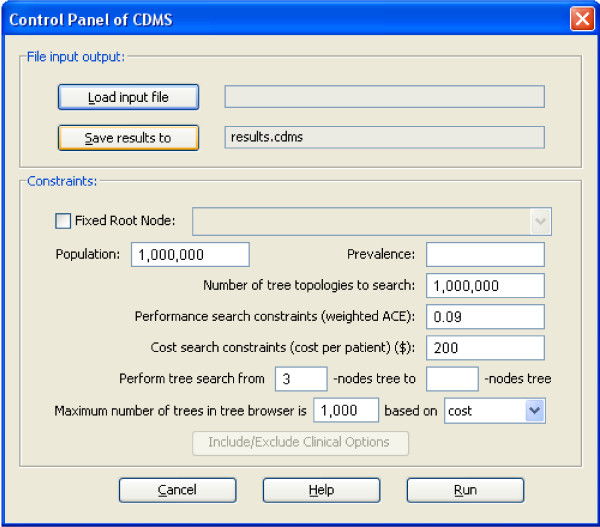
The Control Panel interface of the CDMS.

Running CDMS starts with a *Control Panel *dialog (Figure 1). It is an interface that allows the user to input all necessary information to begin searching. The *Control Panel *includes two parts: the *File Input Output *section, where the user can load the input file and specify the output filename here, and the *Constraints *section, where the user has to input the prevalence value of the type of disease (e.g., cancer) or condition (e.g., drug sensitivity) under study. The prevalence value can be derived from any valid empirical estimate or information resource (e.g., [10], the US national SEER database). The prevalence, and all of the clinical options, should be relevant to the clinical population under study.

All other parameters in this part have default values that the user can modify to suit their study. For example, the user can increase or decrease the values of the population and the number of tree topologies to search.

#### Rooted vs. unrooted searches

The user also can select one clinical option as the fixed root node of the tree by checking the *Fixed Root Node *check box and selecting an option from the drop down list. When this option is activated, CDMS will only search trees that begin with the same clinical option as the first.

#### Including/Excluding Clinical Options

**Figure 2:**
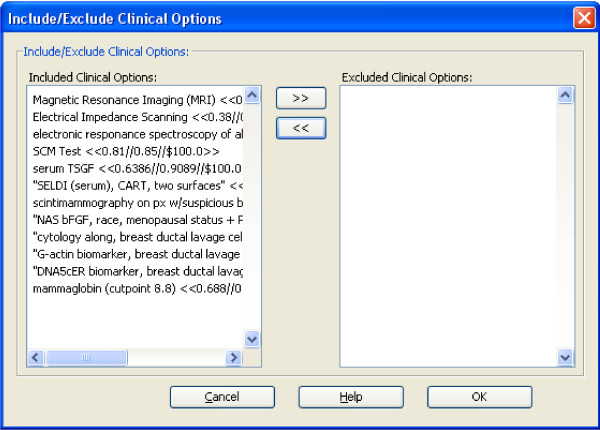
The interface to select clinical options to search for breast cancer.

In some situations, the user may wish to conduct a tree search using only a subset of the clinical options from the input file. In this case, the user can select the options by clicking the *Include/Exclude Clinical Options *button. A subsequent dialog will appear. An example of the dialog is shown in Figure 2. All clinical options are associated with their parameters: SN, SP, and cost. This will allow the user to quickly conduct sensitivity analyses by including two or more instances of options with same name, but with different performance evaluation measures or cost estimate parameters.

#### Stopping a search

The default number of tree topologies to search for all levels of clinical options is 1,000,000. To prevent excessive run times, CDMS has an option to allow the user to intervene at any time prior to completion of the search process. Results generated to that point are shown.

#### Conducting the random tree search

The user starts the random tree searching process by pressing the *Run *button in the *Control Panel *dialog. A progress bar appears and shows the progression of the random tree searching process. The searching process is saved to the *.txt file of same name as the *.cdms file.

## Results

### Use case #1. Breast Cancer Detection Decision Modeling

**Figure 3:**
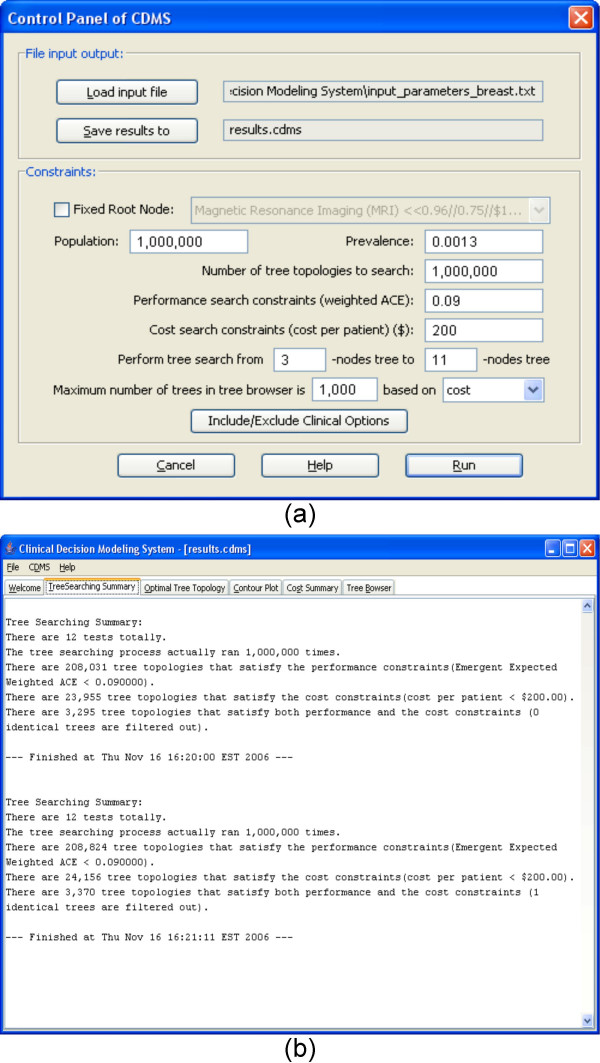
**The input parameters and tree search summary of the CDMS for a trial run for breast cancer**. (a) Control Panel of the run. (b) Tree searching summary.

The input file [see Additional file 2] for Breast Cancer Detection Decision Modeling is in Table 1. The SN and SP estimates were derived from the peer-reviewed literature by JLW. In May 2006, NCBI's Pubmed was searched for abstracts with the keywords "breast cancer" AND "sensitivity" AND "specificity". The first 200 abstracts were read and performance evaluation measures of potentially relevant studies were recorded. Because cost estimates for these newly proposed tests are not yet available, all tests were arbitrarily assigned a hypothetical operational cost of $100. Figure 3(a) shows the parameters that are input using the *Control Panel*. From the Figure, we see that the prevalence for the breast cancer is 0.0013 (The prevalence estimate was obtained from SEER [10] in May, 2006.). All other input parameters are default values. Figure 3(b) shows the tree search summary report. From the summary, we know that there are 12 clinical options (tests) for this search. The second search was performed using 1,000,000 iterations. During the search, 208,824 tree topologies were found that satisfy the performance constraints; 24,156 tree topologies were found that satisfy the cost constraints; and 3,370 tree topologies were found that satisfy both constraints.

**Figure 4:**
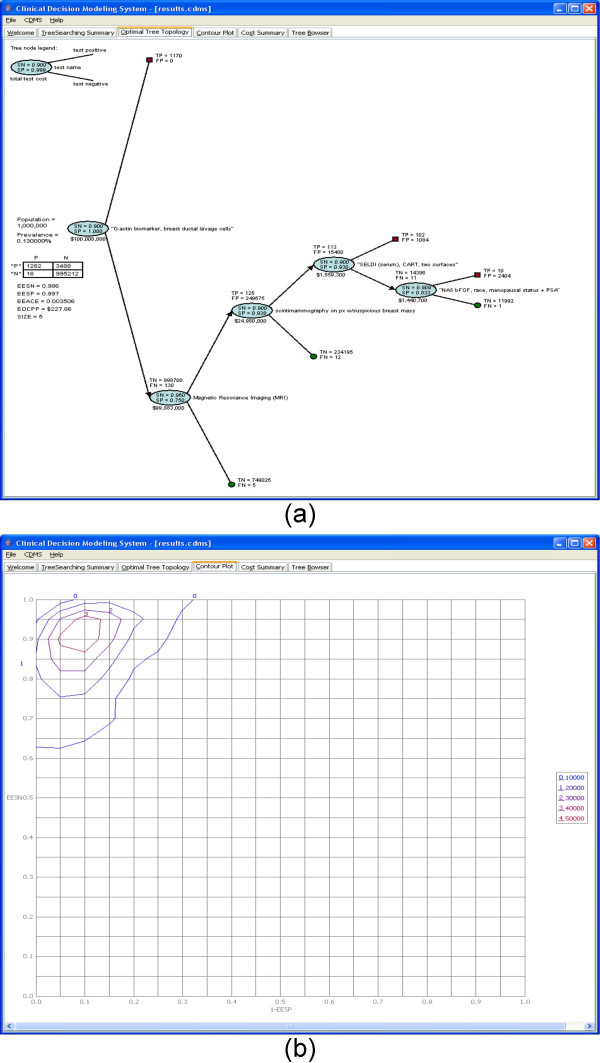
**The optimal tree topology and contour plot of the CDMS for a trial run for breast cancer**. (a) Optimal tree topology. (b) Contour plot.

The *Optimal Tree Topology *tab (Figure 4(a)) shows the best tree topology among the 1,000,000 trees searched for the breast cancer. It is a tree that consists of 5 clinical options. In addition, the confusion matrix for this tree is also displayed. In it, *P *is the number of patients with breast cancer and *N *is the number without breast cancer. Based on the numbers in the confusion matrix, the expected overall performance measure can be calculated. These performance measurements include EESN, EESP, EEACE, and EOCPP.

The *Contour Plot *tab (Figure 4(b)) shows the performance distribution of all 1,000,000 trees searched. Performance measurements for the contour plot are the paired values of EESN and 1-EESP within 2-dimensional bins. For example, if one tree has an EESN = 0.9 and an EESP = 0.85, the coordination in the contour plot is (0.9, 0.15). The contour line in the plot represents a threshold. For example, the contour line of 0 represents 10,000.

**Figure 5:**
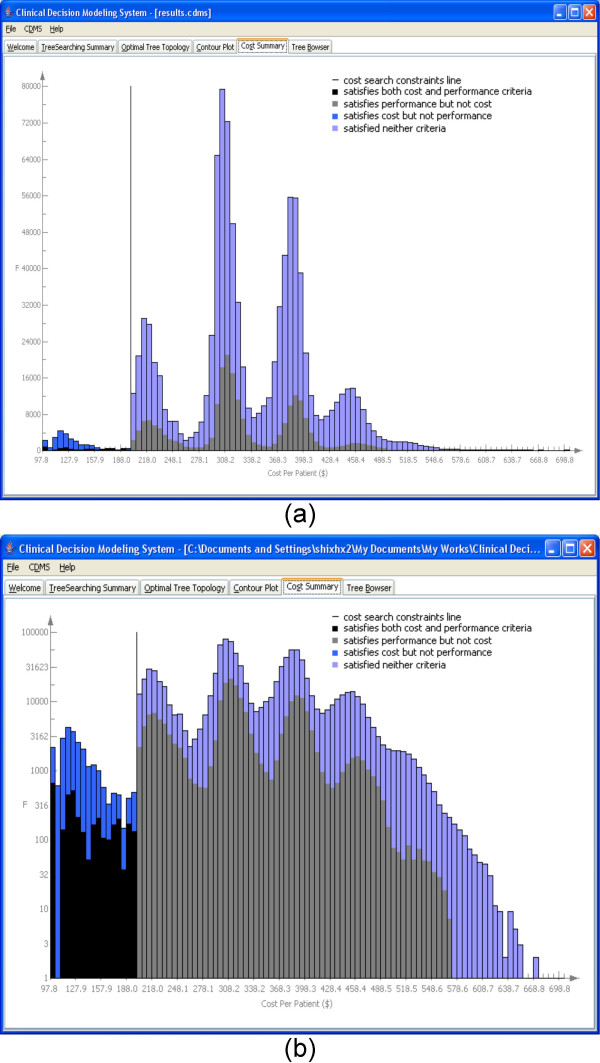
**The cost summary of the CDMS for a trial run for breast cancer**. (a) Normal scale. (b) Logarithmic scale.

The *Cost Summary *tab (Figure 5(a)) shows the distribution of the costs of all the trees searched. The x-axis shows all possible cost values and y-axis is number of counts. A vertical line is added in the distribution to indicate the value of the cost constraints. In the breast cancer, it is $200.00. The cost constraint line is used to distinguish the area that satisfies the cost constraints with the area that does not, under the distribution curve. Different colors are used to incorporate performance information into the cost distribution. To the left of the cost constraints line, darker blue means that all trees satisfy the cost constraints. Within the dark blue bars, black represents the proportion of trees that also satisfy the performance constraints. The user can change to *logarithmic *scales to see these parts more clearly (Figure 5(b)). The user only needs to move mouse above the y-axis and right click. Normal scales and logarithmic scales can be toggled by repeating this operation. To the right of the cost constraint line, light blue represents trees that do not satisfy the cost constraints. Light gray pertains to the proportion of trees that satisfy the performance constraints.

**Figure 6:**
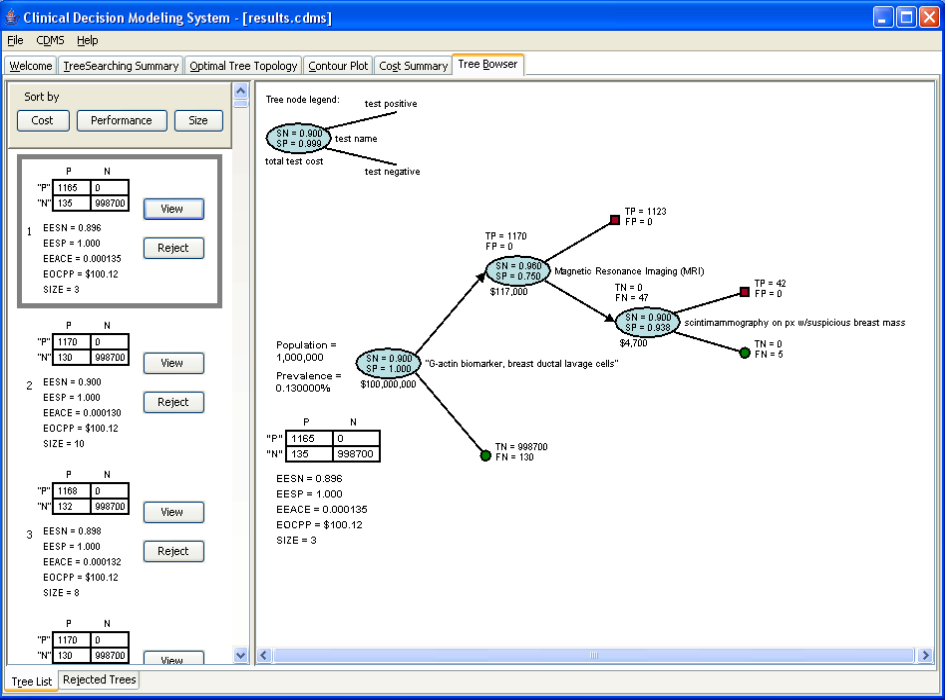
The tree browser of the CDMS for a trial run for breast cancer.

In the *Tree Browser *tab (Figure 6), all tree topologies that satisfy both the performance and cost constraints are listed, but with a size-limitation. In some cases, there may be too many trees that satisfy both performance and cost constraints that computer memory may become limiting. A user-defined upper limit of 1,000 trees can be displayed in the tree browser. The user can sort the trees in the tree browser based on tree's performance (weighted 'emergent expected achieved classification error', or EEACE [see Additional file 1]), cost (EOCPP), size (number of nodes in the tree), or on their own defined optimality criteria (e.g., 'time to test result'; 'risk of harm to patient'). The user also can view each tree graphically by clicking the *view *button. If tree topologies appear nonsensical for practical and ethical clinical workflows, they may be rejected by pushing the *Reject *button. The rejected trees are moved into the *Rejected Trees *sub tab. In the case that some trees are rejected incorrectly after reconsideration, they can be restored back by pushing the *Restore *button. Rejected tree can also be viewed by clicking *View *button in the *Rejected Trees *sub tab.

### Use case #2. Lung Cancer Decision Modeling

**Figure 7:**
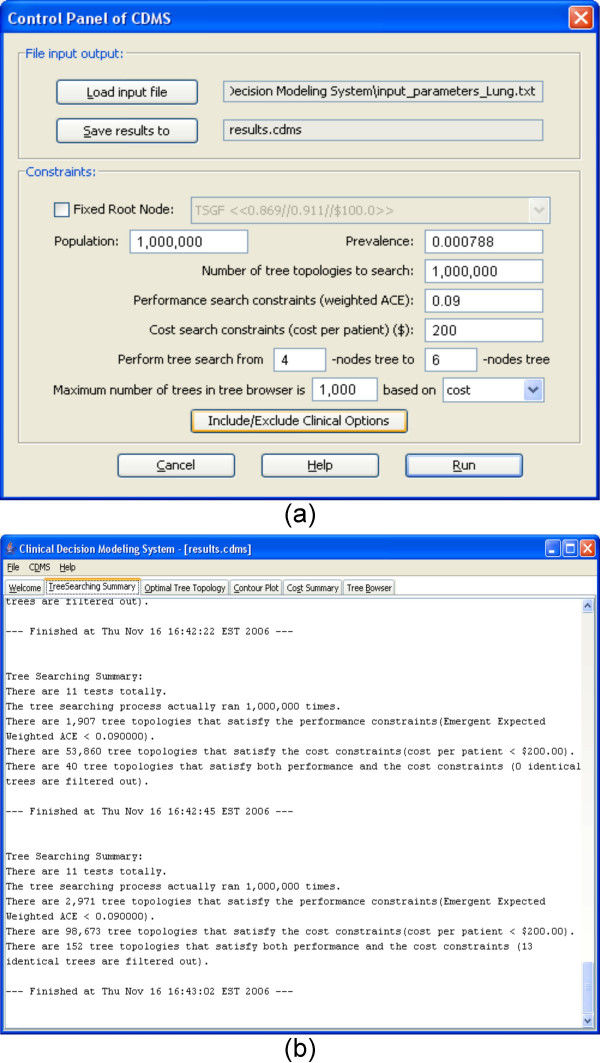
**The input parameters and tree search summary of the CDMS for a trial run for lung cancer**. (a) Control Panel of the run. (b) Tree searching summary.

**Figure 8:**
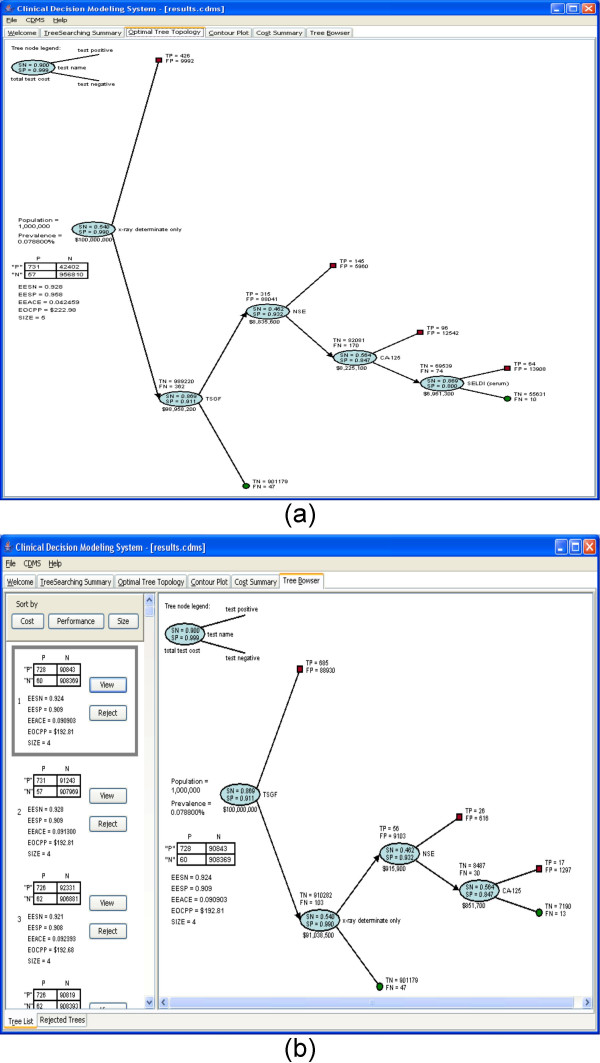
**The optimal tree topology and tree browser of the CDMS for a trial run for lung cancer**. (a) Optimal tree topology. (b) Tree browser.

**Figure 9:**
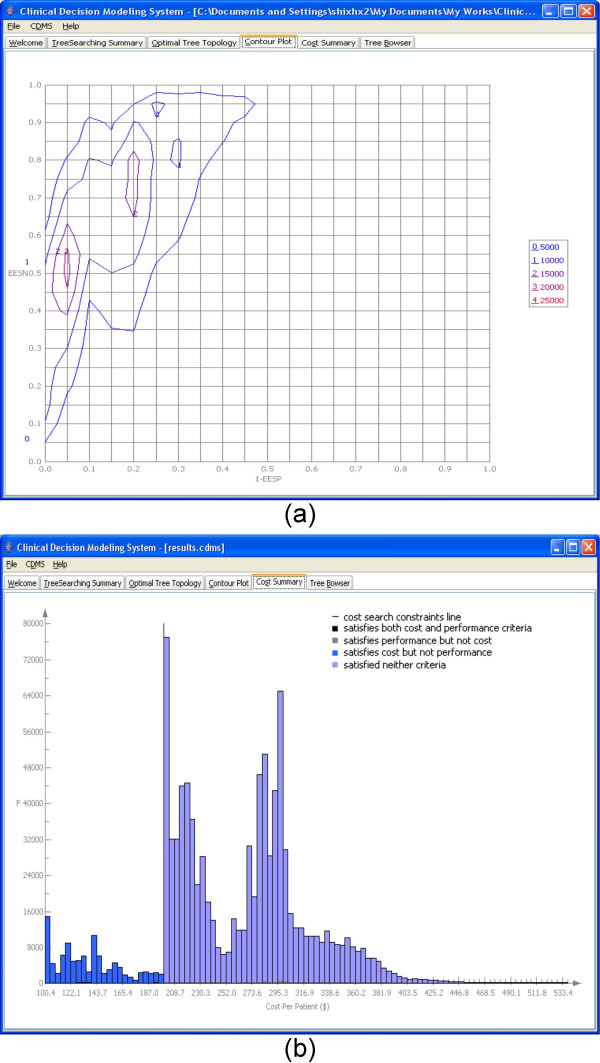
**The contour plot and cost summary of the CDMS for a trial run for lung cancer**. (a) Contour plot. (b) Cost summary.

Figure 7(a) shows the *Control Panel *of our Lung Cancer use case. It includes 15 clinical options (Table 2) in the input file derived from the literature search conducted by JLW. The Pubmed search, conducted in May 2006, terms were "lung cancer" AND "specificity" AND "sensitivity". The first 200 abstracts were read. Due to the small number of applicable reports, an internet search was added, resulting in the finding of a report on three putative biomarkers by Newland Biotech, Inc. Therefore, the *Tree Searching Summary *tab (Figure 7(b)) reports that there are 11 options. The cost of each option was set arbitrarily at $100.00 for this use case. 1,000,000 search iterations were performed. In this case, 4 options are excluded to avoid overly optimistic projections as very high performance potential high-dimensional biomarker sources (e.g., SELDI) require further validation. The most optimal tree topology under the classifier performance criteria, a 5-node tree, is displayed in the *Optimal Tree Topology *tab (Figure 8(a)). From the tab, we can see that the cost per patient of this tree is $222.98, which is greater than the cost constraint ($200.00). That is, the "best" tree does not satisfy the cost constraints. Therefore, it is not included into the *Tree Browser *(Figure 8(b)), which displays only trees that meet both optimality criteria. It is only the "best" tree based on classifier performance. There are 152 tree topologies that satisfy both performance and cost constraints in the third search (Figure 7(b)), among which 13 trees are duplicated. Therefore, only 152 – 13 = 139 trees are listed in the tree browser. CDMS does not display redundant, i.e., identical, trees in the tree browser. As in the breast cancer use case, the *Contour Plot *tab (Figure 9(a)) shows the performance distribution of all 1,000,000 trees searched and the *Cost Summary *tab (Figure 9(b)) shows the distribution of the costs of all the trees searched.

## Discussion

### Major limitation of NDM

All subsequent results are highly dependent on the accuracy and precision of the input performance evaluation, cost and other parameter estimates. Studies should therefore be screened for possible biases, and sensitivity analysis can be conducted to assess the impact of potentially optimistic estimates.

### Major applications of CDMS

The most obvious application of CDMS is the exploration of putative combinations of clinical options for diagnostics. In this application, the idea is to perform a search under the naïve assumption of conditional dependence to minimize searching for pairs or sets of tests for which joint probabilities are needed. Under the assumption of conditional independence, many clinical combinations are likely to be highly optimistic. Importantly, the introduction of conditional dependence however will only lower the EESN or EESP. The exploration of the robustness of specific workflows to conditional dependence can be explored empirically, and acceptable levels of conditional dependence can be determined prior to data collection. In the future, CDMS will allow the user to upload a sparse matrix of conditional probabilities so the calculation of EESN and EESP can be readily modified dynamically using empirically derived conditional probabilities during the tree search as needed.

### Cost-neutral analyses

While the use cases we provide are cost-neutral (assume equal cost of all clinical options, the NDM method implemented by the CDMS software is capable of considering user-provided estimates of cost. Indeed, the default data input format requires cost estimates. It may be useful run a preliminary cost-neutral analysis to determine whether, even under the best-case assumption of conditional independence, any clinically acceptable high-performance combinations exist given the available clinical options. To conduct cost-neutral analyses, the user can specify any common cost for all clinical options (in our use cases, $100).

### Benefits of random tree searching

There are numerous benefits to conducting a random tree search. First, it provides a rapid answer to the question of whether *any *combinations exist that are high-performance; i.e., it answers the question "Does a population of high-performance, cost-effective putative clinical workflows exist?". Second, it allows the manual exploration of near-optimal trees. Leal et al [7] cite a "gap in the literature (that exists) between theoretical elicitation techniques and tools that can be used in applied decision-analytic models". Our approach places the experts (or a committee of experts), in the position of applying their preferences to entire competing decision models based on any number of attributes, both formally included in the valuation, and those inherent to a proposed series of successive clinical steps. Finding the global optimal solution may also not be desirable. In many cases, the theoretically optimal trees may be clinically unacceptable because they are considered impractical, or unethical. As more information about each of the diverse newly proposed tests become incorporated as criteria (e.g., 'risk to of harm to patient'), successive updates to the model searches will become more refined.

### Compatibility with generalized decision modeling

The main core of the NDM search strategy implemented by CDMS is, by design, random tree searching. In the future, additional options that increase overall utility will be added. These include options for automating parameter sensitivity analysis, and it may also include the capability to conduct some critical aspects of standard decision modeling. We view the integrative framework outlined in [5] as a very promising direction to implement our strategy so that each tree search result can be the product of multiattribute functions. For the time being, we foresee applications of the CDMS in the search for ways to integrate diverse sources of clinical data in a manner that allows clinicians to weigh in and discuss and debate their rationale for rejecting specific putative potential workflows, and to identify the critical missing types of information required to finalize decisions needed for highly integrative clinical studies.

### Use by major medical research institutions

Decisions to adopt new clinical options for patient diagnosis and treatment usually follow a hierarchy within an organization, and numerous real-life factors are taken into account. We envision that CDMS might provide impetus for the adoption of clinical options that, when considered in isolation, might not be adopted due to these other factors. Decision-makers at the highest levels in medical research institutions are encouraged to adopt CDMS, and to undertake the team-building exercise of decision modeling. NDM makes the process simple, makes all of the details of all of the factors explicit, and, most importantly, can allow clinical research teams to state the problem of adoption in terms of testable hypotheses (e.g., 'the adoption of clinical workflow *x *will result in a SN of *at least *0.8 and SP of *at least *0.90 at a per-patient cost of *at most *$1300US'), where the hypotheses are based on evidence that the critical pairs of clinical options are, in fact, conditionally independent. This type of research might prove more amenable to expediting translational integration than the traditional 1 vs. 1 (option *x *vs. option *y*) comparisons.

### Scalability

The CDMS software can be used to study the integration of thousands of clinical options; the scalability is limited only by the RAM of the computer used. If the user wishes to consider topologies that include many clinical options, viewing the entire tree may be problematic. In practice, however, most users will likely restrict their consideration to workflows with a reasonable number of options per tree, even when the number of possible clinical options is very large.

### Generalizability

The CDMS software can be used on numerous computational platforms. NDM is a general framework that can be applied to various types of problems in biomedicine, including, for example, integrative diagnostics (as in our use cases), or drug therapy studies when there are multiple choices with conflicting evidence. Modeling the efficacy of various drugs in combination, however, should consider nonlinear dependencies. While CDMS does not yet permit such higher-order dependencies among the clinical options, it could be found useful in helping to focus consideration of alternative combinations of treatments, and their order, considering factors such as cost and accumulated risks associated with negative side-effects.

## Conclusion

It should be recalled that any decision modeling exercise, however implemented, can only ever produce hypotheses that must be tested with empirical data. It is our hope that improvements in the integration of biomarkers for the clinical diagnostics for cancer and other debilitating diseases will be found using CDMS, tested via retrospective studies, updated as needed (for example, as Bayesian networks), and most importantly validated via prospective clinical studies where the decision model is the instrument tested, as a workflow.

## Availability and requirements

**Project name: **Clinical Decision Modeling System

**Project home page: **

**Operating system(s): **Platform independent. Current version tested on Microsoft Windows XP and 2000.

**Programming language: **Java

**Other requirements: **Java JDK or JRE1.5.6 or higher

**License: **(C) 2007 The University of Pittsburgh, All Rights Reserved.

**Any restrictions to use by non-academics: **For commercial licensing, contact The University of Pittsburgh Office of Technology Management (Brian Copple or Marc Malandro, Tel: 412-648-2208).

## Abbreviations

**CDMS**: Clinical Decision Modeling System

**ROC**: Receiver Operating Characteristic

**NDM**: Naïve Decision Modeling

**SN**: Sensitivity

**EESN**: Emergent Expected Sensitivity

**SP**: Specificity

**EESP**: Emergent Expected Specificity

**EOCPP**: Expected Overall Cost Per Patient

**ACE**: Achieved Classification Error

**EEACE**: Emergent Expected Achieved Classification Error

**SEER**: Surveillance Epidemiology and End Results

## Competing interests

Under University of Pittsburgh technology transfer policies, both authors could potentially benefit financially from commercial licenses of the CDMS software.

## Authors' contributions

JLW conceived of this overall approach to decision modeling. HS encoded the algorithms in the CDMS application and designed the random search method. Both authors contributed to the writing of the manuscript and to the generation of the use case results.

## Pre-publication history

The pre-publication history for this paper can be accessed here:



## Supplementary Material

Additional file 1 Appendices. This file includes the *Mathematical Definitions *of some terms used in the paper and a detailed *Random Tree Search Algorithm Description*. Click here for file

Additional file 2 Software. This compressed file includes the software and other necessary files. The software is an executable jar file, 'cdms.jar'. To run it, the user needs an input file. 'input_parameters_breast.txt' is the input file used for the *Use case #1 *in the *Results *section. 'prevalence_breast.txt' includes the prevalence value for breast cancer. 'README.txt' is a text file that includes instructions to run the software. Click here for file
