# Supplementary material for: Clinical decision modeling system
Source: BMC Med Inform Decis Mak. 2007 Aug 13;7:23. doi: 10.1186/1472-6947-7-23 (PMC2131745; doi:10.1186/1472-6947-7-23)
Supplement: Additional file 1 — Appendices. This file includes the Mathematical Definitions of some terms used in the paper and a detailed Random Tree Search Algorithm Description. [file 1472-6947-7-23-S1.doc]

**Appendices**

**A1. Mathematical Definitions**

The calculation of EESN, EESP, EEACE, weighted EEACE, and EECPP are based on the following equations:

$$\begin{matrix} EESN=\frac{\sum_{allLeafNodes} TP}{\sum_{allLeafNodes} TP+\sum_{allLeafNodes} FN} \\ EESP=\frac{\sum_{allLeafNodes} TN}{\sum_{allLeafNodes} TN+\sum_{allLeafNodes} FP} \\ EEACE=\frac{\sum_{allLeafNodes} FP+\sum_{allLeafNodes} FN}{N} \\ weightedEEACE=\frac{\sum_{allLeafNodes} FP+\frac{1-prevalance}{prevalance}\sum_{allLeafNodes} FN}{2N\left(1-prevalance\right)} \\ EECPP=\frac{\sum_{allTestNodes} \left(testCost*\#patiantsDiagnosedByThisTest\right)}{N} \end{matrix}$$

where $N=\sum_{allLeafNodes} TP+\sum_{allLeafNodes} FN+\sum_{allLeafNodes} TN+\sum_{allLeafNodes} FP$ (Total population)

*TP*: True Positive

*FN*: False Negative

*TN*: True Negative

*FP*: False Positive

*Prevalence*: the proportion of cancer patients in the population

**A2. Random Tree Search Algorithm Description**

CDMS currently searches around all possible tree topologies at random. Since there are huge number of possible tree topologies especially when the number of nodes in a tree gets large, and because clinicians should weigh in on the acceptability of the overall workflow, random searching tree topology space is a practical method. It avoids the time-consuming or even implausible complete searching, and it allows manual browsing of near-optimal solutions. Tree topologies are constructed randomly and then the emergent performance and costs per patient are calculated for those trees. Heuristic searches such as branch-and-bound may be added in the future.

Constructing a tree topology starts by picking up a clinical option as the root node. It can be selected randomly by CDMS or specified by the user through the *Control Panel* interface. Therefore, any clinical option could be the root node. Then, another clinical option is randomly picked up and inserted into the tree as the second node. The topology of tree in CDMS is bifurcated. There is the same 50% chance that the second node is put into either one of the two children positions of the root node. After that, the third option is randomly picked up and put into the tree by the general rule described in the next paragraph. This procedure keeps going until a maximum number of nodes are reached. This maximum number is specified by the user through the *Control Panel* interface. The default values for the search range are from the minimum value 3 to the maximum value - total number of the clinical options minus one (n - 1). Certainly, the users can change those two values to any number between 3 and (n - 1) as long as the maximum value is greater than or equal to the minimum value.

The general rule to insert next option into the tree is as follows. Randomly locate a tree node in the existing tree topology. Check if one of its children positions is available. Which child position is checked first is completely randomly. Either one has 50% chance to be checked first. If one of its children position is available, put the new option over there. If both positions are not available, keep searching either one node before (searching up) or after it (searching down) in the array list that holds the tree. The array list holds tree nodes by the order that they enter the tree. Therefore, searching either up or down does not mean to search the tree by the child-parent relation. Searching up or search down is also by 50% chance. Keep searching until there is a child position is available. In the case of searching up, if no position can be found but the searching has reached the root node, change the searching direction and keep searching down the tree topology until a position is available.

The number of possible tree topologies increases extremely fast when number of nodes in a tree increases. In other words, there is much more number of possible tree topologies for large tree than small tree. Therefore, CDMS searched more for large trees than small trees. Specifically, CDMS calculates the number of trees to search for a fixed number of nodes based on an exponential proportion for different size of trees. For example, CDMS searches 5-nodes tree EXP(5)/EXP(3) times as 3-nodes tree. In addition, the user can narrow down the search range by increasing the minimum number of tree or decreasing the maximum number of tree to search through the *Control Panel* interface.

User needs to specify the performance and cost constraints in order to filter out all the trees that do not satisfy both or either of them. The calculation of the emergent expected performance characteristics (sensitivity, specificity, average cost-per-patient) requires an important, initial (Naïve), but testable assumption of independence among the clinical options. The software will help identify critical pairs of clinical options that should, as a priority, be further studied for conditional dependence; thus given all clinical options a sparse joint matrix may ultimately be found to be sufficient.

Planned updates:

1. CDMS will generate and store a ‘critical pairs’ matrix of clinical options for which the assumption of conditional independence should be tested.
2. CDMS will be able to input a joint probability matrix and modify the calculations accordingly.
